# “Understanding Chronic Kidney Disease Self‐Management Barriers and Facilitators: A Consumer‐Led Qualitative Study”

**DOI:** 10.1111/jorc.70055

**Published:** 2026-03-05

**Authors:** Laura E. Lunardi, Lisa A. Matricciani, Richard K. Le Leu, Richard Bastin, David Myers, Merrilyn Bradbrook, David Bradbrook, Rhanee Lester, Effie Johns, Anne Britton, Shilpanjali Jesudason, Shyamsundar Muthuramalingam, Dorothea Dumuid, Paul N. Bennett

**Affiliations:** ^1^ Central Northern Adelaide Renal and Transplantation Service, Central Northern Adelaide Renal and Transplantation Service (CNARTS) Royal Adelaide Hospital Adelaide South Australia Australia; ^2^ College of Health Adelaide University Adelaide South Australia Australia; ^3^ Alliance for Research in Exercise Nutrition and Activity (ARENA), College of Health Adelaide University Adelaide South Australia Australia; ^4^ School of Psychology, College of Education, Behavioural and Social Sciences Adelaide University Adelaide South Australia Australia; ^5^ Central Adelaide Local Health Network (CALHN) CNARTS, Consumer Reference Group Adelaide South Australia Australia; ^6^ School of Nursing and Midwifery Griffith University Queensland Australia

**Keywords:** chronic kidney disease, qualitative research, self‐management

## Abstract

**Background:**

Although self‐management is essential for slowing the progression of chronic kidney disease and improving quality of life, patients continue to face substantial and varied challenges in managing their condition. Existing research has identified barriers to self‐management but less is known about the barriers and facilitators experienced by patients with advanced chronic kidney disease not yet receiving kidney replacement therapy (dialysis and transplantation). Furthermore, few studies have been consumer‐led or have integrated clinician and patient perspectives in a shared discussion environment.

**Aim:**

To explore the barriers to, and facilitators of, chronic kidney disease self‐management from the perspectives of key stakeholders, using a consumer‐led qualitative approach.

**Methods:**

Patients with chronic kidney disease and clinicians were purposively sampled from a large renal service in South Australia to participate in a focus group interview, co‐facilitated by a person living with chronic kidney disease. Three, 2‐h focus group interviews involving 11 renal consumers and six renal clinicians were undertaken following a semi‐structured interview guide that was co‐developed with renal consumers and transcribed verbatim. Transcripts were coded and analysed using inductive thematic analysis.

**Results:**

Six themes emerged: patient individuality, information and education resources, disease and treatment burden, healthcare team services, patient‐clinician relationships, and teaching and learning strategies. Identified barriers included patient passivity, limited chronic kidney disease awareness, fragmented care, impersonal clinical approaches, and physical/emotional distress. Facilitators included positive attitudes, goal setting, trust and satisfaction with clinicians, effective communication, shared decision‐making, person‐centred care and caregiver support.

**Conclusion:**

This study identified that chronic kidney disease self‐management is influenced by interacting personal factors, relational factors and systemic factors. These qualitative insights demonstrate that patients' ability to self‐manage is shaped not only by knowledge, but by emotional burden, confidence, and the quality of relationships within the healthcare system. Consumer‐led approaches that reflect these lived experiences may enhance the relevance and acceptability of future self‐management support.

## 1 Introduction and Background

Chronic kidney disease (CKD) is a major global health condition affecting a growing proportion of the population and is among the leading causes of mortality and morbidity worldwide, with substantial implications for health systems, health‐related quality of life and long‐term care needs (Deng et al. 2025; Evans et al. 2022; Kidney Health Australia 2020; Stenvinkel et al. 2020). In Australia, CKD represents a significant and growing burden on individuals and health services, reflected in rising referrals to specialist renal care and rising demand for long‐term management prior to the initiation of kidney replacement therapies (KRT). The condition imposes a significant day‐to‐day burden on individuals, disrupting daily routines, employment, interpersonal relationships, and emotional wellbeing. As a result, effective self‐management is essential for supporting patient autonomy and enabling individuals to adapt to the ongoing physical and psychosocial challenges associated with CKD (Chen et al. 2011; Devins et al. 2003; Havas et al. 2017).

Self‐management in CKD involves patients' ability to effectively manage symptoms, adhere to prescribed treatment regimes, implement lifestyle adjustments, and better navigate the psychosocial consequences associated with chronic illness (Donald et al. 2018; Lightfoot et al. 2022). Effective self‐management extends beyond access to information and requires the development of knowledge, skills and confidence needed to apply health information in daily life, maintain treatment adherence, and respond appropriately to changes in health status (Dineen‐Griffin et al. 2019; Lightfoot et al. 2022; Lunardi et al. 2023). These attributes, conceptualised as components of patient activation, enable individuals to engage more actively with their healthcare and adapt to the ongoing demands of living with CKD (Lightfoot et al. 2022). Evidence indicates that higher levels of patient activation are associated with improved treatment adherence, more effective symptom monitoring, and delayed disease progression, whereas low activation is linked to poorer health outcomes, often reflecting a reduced capacity to recognise and respond to early signs of health deterioration (Hussein et al. 2021; Lightfoot et al. 2022; Lunardi et al. 2024).

Existing studies identify several barriers that limit patients' engagement in CKD self‐management. These barriers include patients' lack of CKD knowledge and awareness, passive attitude, socio‐economic disadvantage, influence of comorbidities and insufficient patient‐physician communication (Lo et al. 2016; Lopez‐Vargas et al. 2014; Schrauben et al. 2022). Facilitators include trust, good communication and satisfaction with the physician, and family support (Lo et al. 2016; Lopez‐Vargas et al. 2014; Schrauben et al. 2022; Tong et al. 2008). Despite this growing body of evidence, much of the existing research has been externally led and has focused predominantly on the dialysis population. Consequently, there remains limited research exploring what enables and hinders patients not yet receiving KRT to develop their knowledge, skills and confidence required for effective CKD self‐management. In particular, little is known about how individuals in the earlier stages of CKD perceive their self‐management needs or which supports they find most meaningful.

Involving patients living with CKD in research is vital for ensuring relevance, applicability and meaningful translation into practice (Cazzolli et al. 2024; Tong et al. 2008). Engaging consumers with lived experience helps bridge the gap between research and real‐word practice, ultimately improving care and outcomes (Scholes‐Robertson et al. 2024; Tong et al. 2008). Although consumer involvement in CKD research is increasing, patients' perspectives are still not consistently prioritised in the design and conduct of research (Scholes‐Robertson et al. 2024). Qualitative approaches can provide an in‐depth understanding of patients' perceptions and what they consider helpful for promoting healthy CKD self‐management (Guha et al. 2021; Tong et al. 2008). Given the identified gaps, this consumer‐led qualitative study was conducted in collaboration with a Consumer Advisory Group of six people living with CKD who participated in all stages of the research process, from the study design to data analysis and interpretation. It was conducted in South Australia, where renal services are delivered across metropolitan tertiary services and regional settings, with variable access to multidisciplinary and psychosocial support, particularly for patients in earlier stages of CKD (Health A. I. o. and Welfare 2024). Understanding how patients navigate self‐management within this specific health system context is therefore critical to informing locally relevant and transferable care strategies. By integrating consumer and clinician perspectives, this study sought to explore the personal, relational, and organisational factors shaping self‐management capability and to identify gaps in renal care within a tertiary renal service in South Australia.

## 2 Methodology

### 2.1 Study Design and Participants

This study employed an exploratory qualitative design, which allowed a broad investigation through discussion with patients with lived experience of CKD and treating clinicians. An exploratory qualitative design using reflexive thematic analysis (Byrne 2022) was chosen to generate rich, experience‐based insights into CKD self‐management to enable an examination of shared meanings, group dynamics, and the interaction between individual and system‐level factors influencing CKD self‐management. This design was appropriate because the study aimed to explore experiences and perceptions rather than generate theory (as in grounded theory) or focus on lived experience at an individual level (as in phenomenology). A qualitative exploratory approach allowed participants to generate discussion collectively and identify issues that may not emerge in individual interviews.

The term ‘consumer‐led’ we adopted in this study refers to the involvement of members of a Renal Consumer Advisory Group acting as co‐investigators who contributed directly to the study design, refinement of the interview guide, recruitment and co‐facilitation of focus groups. Their involvement ensured that the research questions and interpretations remained grounded in‐patient experience rather than solely in the researcher or clinician perspective. We involved six Consumer Advisory Group members from the Central Adelaide Local Health Network (CALHN) Consumer Partnering and Community Engagement in South Australia, who acted as co‐investigators. This Consumer Advisory Group assisted in the design, recruitment and development of the in‐depth semi‐structured interview guide (Supporting Information S1: Supplementary Material) to conduct three, 2‐h focus group interviews (FG1, FG2, FG3). Consumer co‐facilitation was employed to help minimise any power imbalances and ensure that authentic consumer priorities informed the process. The Consolidated Criteria for Reporting Qualitative Research (COREQ) was used to guide transparent and clear reporting (Supporting Information S1: Supplementary Material for the COREQ checklist) (Tong et al. 2007).

Focus group interviews were conducted from July to September 2024. A purposive sampling method was used aiming to recruit up to 12 renal consumers and six renal clinicians from the largest renal service in South Australia. This focus groups size was considered sufficient to capture diverse perspectives across age, socio‐demographic characteristics and kidney replacement modality consistent with qualitative research standards where depth of insight is prioritised over breadth. The planned sample size (approximately 12 consumers and 6 clinicians) was informed by qualitative methodological guidance indicating that focus groups of four to six participants allow for rich interactional data while enabling diverse viewpoints (Hennink and Kaiser 2022).

Eligible participants were adult English‐speaking with lived experience of CKD and all from a large tertiary Renal and Transplantation unit within South Australia. To ensure diversity, we reviewed participant's characteristics to purposely sample participants with varied age, sex, educational level and stage of their CKD, with or without experience of dialysis and kidney transplant. Renal clinicians were invited to participate from the 60 renal clinicians working with this cohort of patients. Each focus group interview included one Consumer Advisory Group member as a co‐facilitator, four renal consumers and two renal clinicians.

### 2.2 Setting

The focus group interviews were conducted at a tertiary hospital in a metropolitan area, with a video link offered to participants who were unable to attend in person. Consumers who attended the focus groups received refreshments and were reimbursed for time as per the SA Health reimbursement policy with a $35 gift card.

### 2.3 Interviews and Data Collection

Focus groups were conducted using a semi‐structured interview guide (Supporting Information S2: Supporting Material) co‐developed by the Consumer Advisory Group, in collaboration with the research team, to ensure questions were relevant and respectful to participants. The interview guide elicited participants' experiences of living with CKD, including factors that helped and hindered self‐management. Prompts were used to elicit more details and seek clarification. Each focus group was facilitated by researchers (P.B., L.M.) and co‐facilitated by consumer partners (R.B., D.M., E.J.) with four renal consumers and two renal clinicians. Mixed focus groups were intentionally used to allow consumers and clinicians to reflect on one another's perspectives and discuss shared system‐level issues. To minimise potential power imbalances and encourage open sharing, several strategies were employed: co‐facilitators with lived experience led the opening discussion; seating was arranged in a circle without hierarchical positioning; group agreements emphasised confidentiality and respectful listening; and consumer participants were provided with opportunities to speak first during sensitive topics. These strategies aimed to reduce social desirability bias and support psychological safety. To address potential confirmation bias associated with a patient‐centred approach, reflexivity strategies were embedded throughout the study. Data collection and analysis involved both consumers and clinician researchers, enabling peer debriefing and critical reflection on assumptions. Ongoing team discussions were used to challenge interpretations and ensure balanced representation of consumers and clinician perspectives.

No patient‐clinician relationship existed between the interviewer and participants before the study commencement. Each focus group lasted 120 min and was facilitated by researchers (P.B., L.M.) and co‐facilitated by consumers partners (R.B., D.M., E.J.). Lead researcher also took field notes during and immediately after each focus group. These notes captured contextual observations, points of emphasis, and nonverbal dynamics, and were used to support interpretation during coding and theme development. Participant demographic characteristics were recorded.

### 2.4 Data Analysis

A reflexive approach was adopted throughout analysis. Research team members documented assumptions and engaged in regular peer debriefing to discuss how their professional backgrounds (e.g., nephrology research, clinical practice, consumer lived experience) might influence interpretation. These practices supported transparency and helped mitigate potential confirmation bias.

Focus group discussions were audio‐recorded and then transcribed verbatim. Transcripts were de‐identified before analysis and entered into QSR NVivo 14 (QSR International Pty Ltd., Victoria, Australia) before being analysed. NVivo 14 was used to support data management and facilitate comparison of coding across researchers.

A codebook was developed (L.L., P.B., L.M.), who then analysed the transcripts line‐by‐line separately to identify each coherent idea. Analysis was undertaken by following the six stages of thematic analysis (Braun and Clarke 2019). After familiarising with the transcript, the first level coding was independently generated and analysed by L.L. and P.B. (transcripts 1 and 3), and L.L. and L.M. (transcript 2) using an inductive approach. Initial codes were grouped and collapsed into preliminary second‐level coding (subthemes) and refined until agreed upon by L.L., P.B. and L.M. After reading the analysis, all researchers discussed and compared the coding until full agreement was reached and dominant themes developed. Two researchers independently coded 100% of Transcripts 1 and 2, and a third researcher cross‐checked Transcript 3. Coding discrepancies were addressed through interpretive discussion to develop shared reflexive understanding, consistent with a reflexive thematic analysis approach rather than inter‐rater reliability testing.

Member checking was conducted by participants who reviewed theme summaries and commented on accuracy, resonance, and completeness. Consumers confirmed the overall thematic structure and provided clarifications that refined theme wording and interpretive emphasis.

Data sufficiency was monitored iteratively throughout analysis. Saturation was considered reached when no new conceptual insights, codes, or subthemes emerged across the focus group discussions, and when subsequent data repeated previously identified patterns. This decision was made collectively by the analysis team. Descriptive statistics (mean, median and frequency) were used to report demographic characteristics of the study population.

The Lincoln and Guba's criteria (Ahmed 2024) was used to enhance trustworthiness. Credibility was supported through prolonged engagement with participants from the Consumer Advisory Group, mixed focus groups including renal consumers and clinicians, triangulation of consumer and clinician perspectives, and member checking of theme summaries. A transparent audit trail, which includes documented analytic decisions, iterative team discussions, and use of NVivo to manage data systematically was strengthens dependability. Confirmability was supported through reflexive practices, peer debriefing, and the involvement of researchers with diverse disciplinary and lived‐experience backgrounds, ensuring interpretations were grounded in the data rather than individual assumptions. Transferability was addressed through comprehensive descriptions of the study context, participants, and analytic process, enabling readers to judge relevance to other settings.

### 2.5 Ethical Considerations

Before data collection commenced, potential participants were approached in‐person during clinic attendance, over the phone or via e‐mail by the study investigator or clinical staff. Those who expressed interest to participate were then approached by the principal investigator, who informed the study's aims and provided with a participant information sheet for review. All participants provided written informed consent. Participants were explicitly advised of their right to withdraw from the study at any time without any negative consequences. The study was conducted in accordance with the National Statement on the Ethical Conduct of Human (National Statement on Ethical Conduct in Human Research 2023).

## 3 Results

Eleven renal consumers (C) and six renal clinicians (CL) participated in the study. Only one potential participant withdrew prior to participation due to scheduling constraints. Table 1 outlines the demographic characteristics of the renal consumers participating in the focus group. Most consumers were receiving KRT (dialysis or transplant), with one participant not receiving KRT.

**Table 1:** Characteristics of renal consumers participants (*N* = 11).

Variables of renal consumers (*N* = 11)	*n* (%)
Age, year	
Median (range)	46 (33 to 78 years)
Female	4 (36)
Ethnicity	
Australian Indigenous	1 (9)
Australian non‐indigenous	8 (73)
Others (England & Bosnia/Herzegovina)	2 (18)
Current living situation	
With family	9 (82)
Alone	2 (18)
Education	
Below high school	1 (9)
Completed high school	5 (45.5)
Higher than high school (diploma/master)	5 (45.5)
Current kidney treatment modality	
Kidney Transplant	6 (54)
Home Therapies	1 (9)
Haemodialysis in centre	3 (27)
CKD – not receiving KRT	1 (9)

Abbreviations: CKD, chronic kidney disease; KRT, kidney replacement therapy.

Of the six participating renal clinicians, there were two nephrologists, two nephrology nurse practitioners, one CKD nurse and one renal dietitian. They had all been working in kidney care for more than 10 years.

**Figure 1:**
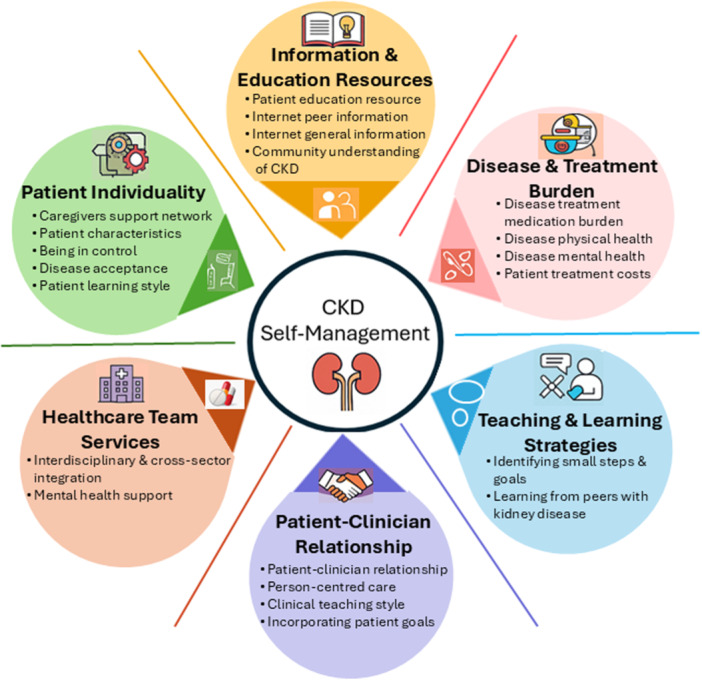
Key themes in CKD management.

As shown in Figure 1, six themes and 18 subthemes were identified to describe what helped and hindered patients in the self‐management of their CKD. These themes capture interacting personal, relational and system‐level influences on self‐management. The themes identified were patient individuality, information and education resources, disease and treatment burden, healthcare team services, patient‐clinician relationship, and teaching and learning strategies. Direct quotes are included to exemplify each theme and subtheme and are summarised in Table 2.

**Table 2:** Barriers and facilitators of self‐management of chronic kidney disease with exemplar quotes.

Themes and definition		Barriers	Facilitators	Subtheme and quote exemplars
Patient individuality	Patient intrinsic factors influencing their behaviours to take an active role to self‐manage their CKD effectively.	Health literacy differences Lack of motivation Passive attitude Limited knowledge and awareness of CKD Printed handout alone	Positive mindset Being in control Personal competence Experiential learning Combined learning materials (app + pamphlet) Caregiver support In‐person education	Patient characteristics
				‘I attribute my good health to a positive attitude and doing what you're told’ ‘You just have to think how much information you give contextualising with all of the other backgrounds of everyone's individual health literacy, their age, their other health conditions’
				Being in control
				‘going down a dialysis path, you've got the whole dialysis team that you can have that back and forth communication but earlier on in that process it's dependent on the doctor to refer you to the dietitian or refer you to different services to get some education’ ‘When I had a transplant, I was in control’
				Disease acceptance
				‘the more you look after yourself, the easier it goes for you’ ‘…people have bad experiences, and that could be part of the reason that they're being put off and not engaged because they don't want to front up to that’
				Patient learning style
				‘Doctors first said to me, oh, don't eat chocolate, you can't eat chocolate. And I was like, ah, what do they know? I'll have some chocolate. Ended up in ED. I didn't realise how serious eating chocolate was’ ‘App's are good in between time if you've got your nephrologist appointment in two weeks’ time but you've got a bit of a question that isn't an emergency situation, you could get a little bit of information around it and then flag it within nephrologist’ ‘I couldn't have learned that from a pamphlet’
				Caregivers support network
				*‘*Your family, the people you care for are great motivators to keep you on the track’ ‘I pretty much self‐manage it all on my own. It would be nice to have family close by, but I don't have that option unfortunately’
Information and education resources	Learning, teaching, and research materials in any format and medium in the public domain that influence patients' knowledge of kidney disease	Unrelievable information (outdated) Lack of community understanding of CKD Impersonalised approach	Kidney consumer website Online peer support	Patient education resource
				‘I think pamphlets and stuff a bit of a waste, whereas even if it's just in the email, you can always get back through an email, even if it's months ago you can search up kidney health and it would come up in your search results, whereas if you have a pamphlet and you put it anywhere, it's just going to go in the bin eventually’. ‘That folder has sat in the cupboard, and I've never got it out since. I don't want to read this black and white folder that's cheaply printed, and I get it, everyone's time constraint, but that's just wasting their time and my time’
				Internet peer information
				‘I did a bit of self‐discovery with Instagram and once you filter through what's real and what's not and I've ended up connecting to about a thousand people. And most of the talk is less physical and more how are you coping’
				Internet general information
				‘Kidney Health Australia or Transplant Australia, I think are quite useful’ ‘Some of the nutrition information, for example, is quite outdated’
				Community understanding of CKD
				*‘people look diabetes, and hypertension is big in the community. Yeah. That's huge. But we don't get much information about kidney health’*
Disease and treatment burden	Disease‐related factors inherent to CKD progression to influence self‐management and health activities	Disease effect on physical wellbeing Psychological stress to cope with CKD progression Adverse effect of medications Overwhelming effect of diet/fluid restriction Cost of treatment Perceived lack of financial support by the health services		Disease physical health burden
				*‘*Before I was on dialysis, I was a sponsored athlete. Kidney disease has taken it away from me. If it's not with the fistula in the arm, it was the catheter in the chest and then it's not being able to sleep for nights. Then negative damage my legs. So now I can't run at all even to save my own life. I had to pretty much rediscover hobbies because it took every one of my lifestyles away’.
				Disease mental health burden
				*‘I'd be driving to work some days going, that's a song they're going to play at my funeral. That's, that's the type of emotion that I had’* *‘you're in your own sense of suffering, you're more aware of it, you're aware of another layer, which is less medical and more mental’*.
				Disease treatment medication burden (medication, diet, fluid allowance)
				*‘*…*the side effects still with the drugs that you have to use cause neuropathy. I can't really walk as far as you see, which cause me a lot of frustration’* *‘When I was first in hospital, they measured how much I drank in a day, and I drank six litres of water and that was a normal day for me. And then they cut me down to a litre and a half. Then, I've had a few instances where my potassium's been sky high, you think you can eat something and it's one of those things that I ate just last week so I can eat it again and then, I end up in ed’*
				Patient treatment costs
				*‘There's a high‐cost burden with all the dialysis and the associated medications and stuff and transport and car parking’* *‘Medication finances is a really big thing, and I believe that the doctors and the politicians should give a healthcare card to those that have got chronic illnesses and have lots of medications and everything. And then you would probably find that the compliance of tablets will be more forthcoming.’*
Healthcare team services	Patients' extrinsic factors caused by healthcare services and clinicians with distinct roles.	Incoordination of care Fragmented healthcare service Lack of mental health support in CKD		Interdisciplinary and cross‐sector integration
				*‘it is quite uncoordinated from a patient point of view at times, like it is a bit disconnected’* *‘oh my God, none of you guys talked to each other, it's hilarious’* ‘*the only thing I found frustrating in the whole thing is the disconnect between the private health system and the public health system*
				Mental health support
				*‘prioritizing my kidney health and mental health, which are kind of connected’* *‘I've had several different episodes throughout my kidney disease pathway. It would've been useful for health professionals to actually talk about the mental health side of things, but it was not’* *‘there's not necessarily a focus on mental health’* *‘maybe you needed more referrals for emotional support maybe or that type of counselling that’*.
Patient–clinician relationship	Influencing factors that affect clinicians‐patient communication, rapport and trust positively or negatively	Inefficient patient‐clinician communication Clinician empathy Didactic health advice Inconsistent information	Patient satisfaction/trust in clinicians Effective communication Person‐centred care approach Clinician availability	Patient–clinician relationship
				*‘Self‐management and making sure someone feel empowered is all about shared decision making’* *‘If they said to me, go and stand in that corner for 20 min after every meal, I go and stand in the corner for 20 min. You got to trust that the people around you that are giving you professional advice’*
				Person‐centred care
				*‘They care about me as the person, not me as a patient’* *‘what worked for me might be completely opposite for someone else’* *‘Some of them are just so dry and it's hard to have a genuine conversation’*
				Clinical teaching style
				*‘it's how we can give that consistent message to help support people through that journey is probably one thing that I see’* *‘a lot of the patients get bit dazzled with all the jargon and they need to ask what all this means’* *‘this person's just telling me not giving me an opportunity to explain, speak and questions’*
				Incorporating patient goals
				*‘and having patients bringing their expertise, which is what your life goals and values are, and then working with doctors and health professionals who have expertise in evidence‐based medicine and working together to get the best outcome’* *‘It makes people feel in control of whatever decision they come to; that's much more effective than sort of the old school way of telling people what to do’*
Teaching and learning strategies	Patients' perception of clinician influencing factors that promote critical and reflective thinking, skills and confidence to self‐manage their CKD	Clinician lack of time Clinician lack of responsiveness Use of jargon Feeling overwhelmed with information	Including patients’ healthcare goals Individualised approach Breaking down barriers Tailored education Interacting with peers Staff competence	Identifying small steps and goals
				*‘when they can break it down to a level that I understand, that's when I found my outcomes become a lot better’* *‘One of the things that I've learned to do or make a point of doing is asking people what's important to them’* *‘Just going out and doing things that I like doing, like going movies or going out with friends’*
				Learning from peers with kidney disease
				*‘…it's not another brochure, it's got to be a real‐life story, it's got to feel real and authentic’* *because I would've had someone else to talk to bounce, you know exactly what you're saying’*

The results are presented using a consistent qualitative analytic structure, with each theme defined, supported by illustrative data excerpts, and followed by analytical commentary explaining how the data demonstrate the identified findings.

### 3.1 Theme 1: Patient Individuality

Patient individuality refers to a person's intrinsic factors influencing their behaviours to take an active role to self‐manage their CKD effectively. This theme highlights how patients' attitudes, experiences, learning preferences and social support shape their capacity to manage CKD. Five subthemes were identified: patient characteristics, being in control, disease acceptance, patient learning style, and caregiver support network.

#### 3.1.1 Patient Characteristics

Some participants expressed the importance of a positive mindset to help self‐manage their kidney disease and maintain their well‐being avoiding negative thoughts ‘I attribute my good health to a positive attitude and doing what you're told’ (C3‐FG2) and ‘…not focused on too much of the bad things’ (C3‐FG1) were examples of this. These accounts suggest that optimism and acceptance may support ongoing engagement with health behaviours. One clinician highlighted the importance of tailoring information to patient demographics, health literacy and clinical characteristics by stating ‘You just have to think how much information you give contextualising with all of the other backgrounds of everyone's individual health literacy, their age, their other health conditions’ (C1‐FG3). Clinicians often framed individuality in terms of health literacy, comorbidities, and information‐processing capacity, whereas consumers described individuality more in terms of personal mindset and emotional coping. This contrast shaped how each group understood the barriers to self‐management.

#### 3.1.2 Being in Control

The theme of being in control of one's health involved the participants' proactive choices and actions to maintain or improve a healthy lifestyle. However, patients noted that opportunities to exercise control varied depending on their stage of kidney disease and treatment modality. Those receiving KRT described having more opportunities for being in control and communication with the healthcare team compared with those not yet receiving KRT *‘…*going down a dialysis path, you've got the whole dialysis team that you can have that back and forth communication but earlier on in that process it's dependent on the doctor to refer you to the dietitian or refer you to different services to get some education*’* (C1‐FG3). These accounts demonstrate that perceived control was strongly linked to treatment modality and service structure. Patients on KRT described regular interactions with staff that increased their confidence and sense of agency, whereas those earlier in their CKD journey reported limited access to multidisciplinary support, shaping a reduced sense of control. In contrast, another patient expressed ‘*When I had a transplant, I was in control*’ (C4‐FG1) as the only time he felt truly in control of his health.

#### 3.1.3 Disease Acceptance

Disease acceptance involves adapting to their kidney disease, acknowledging its limitations, and finding ways to integrate their CKD into their life while maintaining a positive attitude. Many participants shared that living with CKD over time helped them better manage the condition and come to terms with its limitations. All participants recognised that self‐management is crucial to maintain or improve their well‐being ‘the more you look after yourself, the easier it goes for you’ (C1‐FG3). However, several participants explained their lack of knowledge of their CKD trajectory and its limitations, which often led to negative thoughts and emotions hindering them from engaging in self‐management behaviour ‘…people have bad experiences, and that could be part of the reason that they're being put off and not engaged because they don't want to front up to that’ (C3‐FG2). This suggests that emotional readiness and understanding are critical components of effective self‐management.

#### 3.1.4 Patient Learning Style

Patients have different ways to learn how to look after their health; some participants did not like printed handouts ‘I couldn't have learned that from a pamphlet’ (C3‐FG1) while others prefer the use of apps “…apps are good in between time if you've got your nephrologist appointment but you've got a bit of a question that isn't an emergency situation, you could get a little bit of information around it and then flag it within nephrologist” (C3‐FG3). Some learnt the importance to self‐management of their health through a life‐threatening experience ‘doctors first said to me, oh, don't eat chocolate, you can't eat chocolate. I'll have some chocolate. Ended up in ED. I didn't realise how serious eating chocolate was’ (C3‐FG3). These findings indicate that flexible, multimodal education approaches are needed to accommodate individual learning styles.

#### 3.1.5 Caregiver Support Network

Patients expressed the valuable emotional and psychological support from their caregivers (family/friends) in helping and motivating them to manage their health. Their support and supervision helped patients solve their health problems that arose during the illness trajectory ‘your family, the people you care for are great motivators to keep you on the track’ (C1, FG2). The absence of social support hindered them from self‐managing their healthcare: ‘I pretty much self‐manage it all on my own. It would be nice to have family close by, but I don't have that option unfortunately’ (C3‐FG3). This underscores the role of social support in sustaining self‐management behaviours.

### 3.2 Theme 2: Information and Education Resources

Information and education resources refer to learning, teaching, and research materials in any format and medium in the public domain that influence patients' knowledge of kidney disease. This theme describes how patients and clinicians evaluate the availability, usefulness, and personal relevance of CKD information. It reflects how resource format, clarity, currency, and accessibility shape patients' ability to understand and act on health advice. This theme comprised four subthemes or categories: patient education resources, internet peer information, internet general information, and community understanding of CKD.

#### 3.2.1 Patient Education Resources

Participants expressed that paper‐based information itself is not helpful in self‐managing their CKD ‘I think pamphlets and stuff are a bit of a waste, whereas even if it's just in the email, you can always get back through an email, even if it's months ago you can search up kidney health and it would come up in your search results, whereas if you have a pamphlet and you put it anywhere, it's just going to go in the bin eventually*’* (C3‐FG3) and ‘That folder has sat in the cupboard, and I've never got it out since. I don't want to read this black and white folder that's cheaply printed, and I get it, everyone's time constraint, but that's just wasting their time and my time' (C3‐FG1) are examples of this. These findings indicate a mismatch between educational delivery methods and patient preferences.

#### 3.2.2 Internet Peer Information

Participants reported that social networks help because they can learn from the lived experiences of others, especially in terms of coping mechanisms. However, there are biases because patients have a different lived experience with CKD ‘I did a bit of self‐discovery with Instagram and once you filter through what's real and what's not and I've ended up connecting to about a thousand people. And most of the talk is less physical and more how are you coping’ (C4‐FG1). While peer information was supportive, participants acknowledged variability in experiences, highlighting both the value and limitations of informal online networks.

#### 3.2.3 Internet General Information

Kidney health online resources were discussed as relevant educational materials ‘Kidney Health Australia or Transplant Australia, I think are quite useful’ (C1, FG1). Those materials are useful and easy to access wherever the patients are, but they are not personalised and can sometimes be obsolete, as remarked by a clinician: ‘Some of the nutrition information, for example, is quite outdated’ (CL2, FG2). This suggests that while online resources are accessible, they require regular updating and contextualisation.

#### 3.2.4 Community Understanding of CKD

Participants expressed that the understanding of kidney disease in the community is often limited, especially in disadvantaged communities such as First Nations people where CKD is highly prevalent. This sentiment was capture in the following statement: ‘…people look diabetes and hypertension is big in the community. That's huge. But we don't get much information about kidney health’ (C1‐FG1) These accounts point to broader social factors influencing self‐management.

### 3.3 Theme 3: Disease and Treatment Burden

Disease and treatment burden refers to disease‐related factors inherent to disease progression as a barrier to engaging in healthy activities. This theme captures the physical, psychological, and practical impacts of CKD and its treatments that inhibit patients' capacity to self‐manage. Burden was described not only as symptom‐related but as an accumulation of disruptions across daily life, identity, work, finances, and relationships. Four overarching subthemes were found: disease physical health burden, disease mental health burden, disease treatment burden (medication, diet, fluid allowance) and patient treatment costs.

#### 3.3.1 Disease Physical Health Burden

Participants perceived CKD physical symptoms negatively impacted their daily activities hindering them from self‐managing their health and making them feel like they were losing their autonomy. This sentiment is captured in the following statement: ‘Before I was on dialysis, I was a sponsored athlete. Kidney disease has taken it away from me. If it's not with the fistula in the arm, it was the catheter in the chest and then it's not being able to sleep for nights. Then negative damage my legs. So now I can't run at all even to save my own life. I had to pretty much rediscover hobbies because it took every one of my lifestyles away’ (C3‐FG3). These experiences illustrate how declining physical capacity can undermine self‐management.

#### 3.3.2 Disease Mental Health Burden

Participants reported the effect of dialysis initiation making them feel overwhelmed by emotions such as denial, anger, depression, frustration and fear of the unknown. This was echoed in the following quotes: ‘I'd be driving to work some days going, that's a song they're going to play at my funeral. That's, that's the type of emotion that I had’ (C3‐FG1) and ‘…you are in your own sense of suffering, you're more aware of it, you're aware of another layer, which is less medical and more mental’ (C4‐FG1). These findings emphasise the close relationship between mental health and self‐management capacity.

#### 3.3.3 Disease Treatment Burden (Medication, Diet, Fluid Allowance)

A patient's ability to manage their symptoms is more effective when the symptoms are better controlled; however, the adverse effect of some medications or difficulties in adjusting the diet and fluid allowance can negatively aggravate or add new symptoms, negatively impacting their daily activities and hindering them from self‐managing their health ‘…the side effects still with the drugs that you have to use cause neuropathy. I can't really walk as far as you see, which cause me a lot of frustration’ (C2‐FG3). ‘When I was first in hospital, they measured how much I drank, and I drank six litres of water and that was a normal day for me. And then they cut me down to a litre and a half. Then, I've had a few instances where my potassium's been sky high, you think you can eat something and it's one of those things that I ate just last week so I can eat it again and then, I ended up in ED*’* (C3‐FG3). Participants recounted frustration and adverse events resulting from treatment complexity, illustrating how treatment demands can compound disease burden and hinder self‐management.

#### 3.3.4 Patient Treatment Costs

Treatment costs related to CKD were a barrier to adhering to medical recommendations among patients ‘There's a high‐cost burden with all the dialysis and the associated medications and transport and car parking’ (CL1‐FG3) as expressed by an interviewed clinician. While a patient expressed ‘Medication finances is a really big thing, and I believe that the doctors and the politicians should give a healthcare card to those that have got chronic illnesses and have lots of medications and everything. And then you would probably find that the compliance of tablets will be more forthcoming’ (C1‐FG3). Participants suggested that financial support could improve adherence and outcomes.

### 3.4 Theme 4: Healthcare Team Services

Healthcare team services refer to healthcare service factors that help or hinder patients' self‐management behaviours. This theme describes how the organisation, coordination, and responsiveness of healthcare services influence patients' ability to self‐manage. It reflects both consumer experiences of fragmentation and clinician recognition of system constraints. This theme comprised two subthemes: interdisciplinary and cross‐sector integration, and mental health support.

#### 3.4.1 Interdisciplinary and Cross‐Sector Integration

Ineffective communication between clinicians and patients emerged as a key factor that hindered patients from trusting the health system and engaging with them to take more proactive action to self‐management their CKD. This point is echoed in the following quotes: ‘It is quite uncoordinated from a patient point of view at times, like it is a bit disconnected’ (C1‐FG1) and ‘…oh my God, none of you guys talked to each other, it's hilarious’ (C4‐FG1). A lack of coordination and unresponsiveness within the healthcare system was reflected in this quote ‘I found frustrating the disconnection between the private health system and the public health system’ (C3‐FG2). These accounts highlight how lack of coordination undermines confidence and continuity of care.

#### 3.4.2 Mental Health Support

Participants expressed the detrimental effect of their CKD progression and dialysis initiation on mental health and the lack of mental health support. Participants remarked on the importance of prioritising mental health support and for renal services to routinely monitor the mental status of their patients, ‘Prioritising my kidney health and mental health, which are kind of connected’ (C4‐FG1). But the mental health support received in the renal service is limited I've had several different episodes throughout my kidney disease pathway. It would've been useful for health professionals to actually talk about the mental health side of things, but it was not’ (C3‐FG1). This indicates a gap in routine service provision.

### 3.5 Theme 5: Patient–Clinician Relationship

Patient‐clinician relationship refers to influencing factors that affect health professional‐patient communication, rapport and trust positively or negatively. This theme highlights relational factors shaping communication, trust and engagement. This theme comprised four subthemes: patient‐clinician relationship, person‐centred care, clinical teaching style and incorporating patient goals.

#### 3.5.1 Patient–Clinician Relationship

The patient–clinician relationship, built on respect, trust, and open communication, was described as crucial for engaging patients in more positive self‐care behaviour. This is illustrated by the following quotes: ‘Self‐management and making sure someone feel empowered is all about shared decision making’ (CL1‐FG1) and ‘If they said to me, go and stand in that corner for 20 min after every meal, I go and stand in the corner for 20 min. You're going to trust that the people around you that are giving you professional advice’ (C3‐FG2). Both clinicians and consumers described trust and shared decision‐making as central to effective self‐management, underscoring the influence of relational trust.

#### 3.5.2 Person‐Centred Care

Participants described the importance of person‐centred care to enable self‐management of their CKD ‘They care about me as the person, not me as a patient’ (C2‐FG1). However, this approach needs to be tailored to the patient's characteristics and needs ‘What worked for me might be completely opposite for someone else’ (C3‐FG3). Patients also expressed frustration when clinicians do not show empathy or listen to the patients ‘Some of them are just so dry and it's hard to have a genuine conversation’ (C3‐FG3). Consumers emphasised the emotional qualities of the relationship (feeling heard, respected), while clinicians focused on communication consistency and clarity. This divergence reflects different priorities, but both recognise the relational foundation of effective self‐management support.

#### 3.5.3 Clinician Teaching Style

A clinician's teaching style focuses on the practical, real‐world application of knowledge and skills to enable patients to understand their disease and its symptoms, self‐manage their condition, and when to seek medical advice. To enable patient active engagement in their healthcare, there is a need to provide clear and consistent instructions ‘…it's how we can give that consistent message to help support people through that journey is probably one thing that I see’ (CL1‐FG3) and ‘A lot of the patients get a bit dazzled with all the jargon and they need to ask what all this means’ (C2‐FG3) are examples of this. However, a lack of responsiveness from some clinicians hinders patients to engage in the learning process ‘This person's just telling me not giving me an opportunity to explain, speak and questions’ (C1‐FG1). These accounts illustrate how complex language and unresponsive communication can hinder learning and self‐management.

#### 3.5.4 Incorporating Patient Goals

Setting clear goals, developing effective strategies and engaging patients in problem solving are crucial to engage patients to take control of their health. This approach was acknowledged by patients and clinicians from participating in this study ‘*…*and having patients bringing their expertise, which is what your life goals and values are, and then working with health professionals who have expertise in evidence‐based medicine and working together to get the best outcome’ (CL1‐FG1) and ‘It makes people feel in control of whatever decision they come to; that's much more effective than sort of the old school way of telling people what to do’ (CL2‐FG1). Both consumer and clinicians emphasised the importance of aligning care with patients' personal goals, describing goal setting as empowering and more effective than directive approaches.

### 3.6 Theme 6: Teaching and Learning Strategies

Teaching and learning strategies refer to influencing factors that promote critical and reflective thinking and skills, helping patients take positive action to protect, enhance, and advocate for their healthcare and well‐being. While Theme 1 focuses on individual readiness and Theme 2 on informational resources, this theme addresses how learning is facilitated through approaches such as breaking information into manageable steps, tailoring advice, and using peer learning. This theme comprised two subthemes: identifying small steps and patients' goals and learning from peers with kidney disease.

#### 3.6.1 Identifying Small Steps and Patient's Goals

Clinicians and patients can agree on small, realistic steps toward healthier behaviours, helping to avoid overwhelming patients and to set them up to succeed ‘when they can break it down to a level that I understand, that's when I found my outcomes become a lot better” is an example of this’ (C2, FG1) illustrated this. Clinicians also described asking what matters to patients ‘One of the things that I've learned to do or make a point of doing is asking people what's important to them’ (CL2‐FG2). A consumer described motivation linked to maintaining valued activities ‘Just going out and doing things that I like doing, like going movies or going out with friends’ was expressed by a consumer' (C3‐FG1). These accounts show how breaking behaviours into manageable steps and aligning care with individual goals can reduce feelings of being overwhelmed and support sustained engagement.

#### 3.6.2 Learning from Peers With Kidney Disease

Participants expressed the positive impact of peer support on improving their knowledge and skills to self‐manage their CKD: ‘…it's not another brochure, it's going to be a real life story, it's going to feel real and authentic’ (C4, FG1) and ‘*…*because I would've had someone else to talk to bounce, you know exactly what you're saying’ (C2, FG1). Peer learning was described as authentic, relatable and emotionally supportive. Participants described valuing learning from others with lived experience rather than formal educational materials alone.

Together, these themes demonstrate that CKD self‐management is shaped by interacting personal, relational and system‐level factors, providing the foundation for the interpretive discussion that follows.

## 4 Discussion

This study aimed to explore barriers and facilitators to CKD self‐management by bringing together the perspectives of patients and clinicians in a consumer‐led, mixed‐stakeholder focus group format. The findings reveal how individual, relational, and systemic factors interact to influence patients' knowledge, skills, and confidence, core components of patient activation. While previous research has identified a range of barriers and facilitators to CKD self‐management, less is known about how consumers and clinicians jointly understand and negotiate these influences within routine renal services, as informed by participants' retrospective reflections on earlier stages of their CKD journey when they were not yet receiving KRT. In the Australian context, where access to multidisciplinary and psychosocial supports can vary, this consumer‐led, mixed‐stakeholder approach provides new insight into how system structures, communication practices, and patient readiness interact to shape self‐management capability.

Focus groups, facilitated by consumer researchers demonstrated that CKD self‐management is shaped by interacting facilitators and barriers across emotional, informational, relational, and system‐level domains. Key facilitators included a positive mindset, a sense of control, caregiver support, patient satisfaction and trust in clinicians, effective communication, a patient‐centred care approach, and tailored education. Key barriers included limited knowledge and awareness of CKD, passive attitudes, the burden of disease and treatment, psychological distress related to disease progression, uncoordinated care, lack of mental health support, and ineffective communication with clinicians. Importantly, participants described these influences as dynamic and mutually reinforcing rather than isolated factors. By adopting a consumer‐led qualitative approach where consumers informed the development and delivery of questions to participants, this research treated patients as partners in knowledge generation, straightening relevance and applicability of findings for clinical practice.

Our study provides insight into the perspectives of patients with advanced CKD, their information priorities and reasons underpinning their preferences to improve their knowledge, skills and confidence (patient activation components) to optimise their self‐management. Patient individuality, for example, aligns with the activation domains of knowledge, skills, and confidence, where low health literacy or passive attitudes indicate limited activation, while a positive mindset, perceived control and experiential learning reflect higher activation. Similarly, barriers such as treatment burden and fragmented care can be understood as factors that interfere CKD patients' ability to translate knowledge into confident self‐management behaviours. Conversely, facilitators such as shared decision‐making and person‐centred communication are strategies that might enhance activation by increasing patients' perception of empowerment and control over their healthcare. Explicitly situating each theme within the patient activation approach highlights that interventions need not only to address surface‐level barriers but also to build the underlying capacity required for sustained self‐management behaviour.

We found that recognising and responding to patient individuality via tailored self‐management programs is important to facilitate active engagement in CKD care. This has also been demonstrated in prior studies that show that personalised approaches are needed to enable patients to take an active and effective role in their healthcare (Lightfoot et al. 2022). Such tailoring is especially important for patients who have a passive attitude towards their health management, as is often observed among older or vulnerable populations (Fracso et al. 2022). Our study highlighted passivity as a key barrier that limits patients' acquisition of the knowledge and skills necessary for self‐management, consistent with previous research (Hwang et al. 2020; Lopez‐Vargas et al. 2014). An individualised strategy should begin by assessing a patient's level of engagement and, where necessary, focus on increasing their activation and capacity for self‐management. An individualised approach also needs to recognise the extent to which patients feel overwhelmed by the cumulative burden of symptoms, treatments, and appointments, as well as understanding the availability and strength of social support systems (family/friends). Tailoring care to these characteristics can help bridge support gaps or leverage existing networks to encourage and sustain patient self‐management. Participants described self‐management as a dynamic and relational process shaped by personal readiness, emotional regulation, system navigation, and clinician communication. A notable and somewhat unexpected finding was how strongly treatment modality and service structure shaped participants' sense of control, with consumers describing substantially more access to education and support after commencing KRT than earlier in their CKD journey.

Emotional burden emerged as a central influence on self‐management capability, with fear, grief, and uncertainty reducing cognitive capacity to process information or adopt recommended behaviours. This supports growing evidence that psychological distress undermines self‐management and highlights the need to integrate mental health support within CKD models of care rather than treating it as an adjunct (Cardol et al. 2023; Escudero‐Lopez et al. 2024; Schmill et al. 2025). Consistent with recent studies, caregiver support was described as alleviating distress and enhancing patients' capacity to adhere to self‐management behaviours, reinforcing the importance of recognising patients' social context when designing interventions (Escudero‐Lopez et al. 2024; Gao et al. 2025). These findings highlight the importance of recognising each patient's unique circumstances throughout their CKD self‐management journey.

Our study found that the availability of information and resources about CKD was identified as another important factor influencing patient self‐management. Participants expressed limited availability of CKD prevention information within the community, noting that both paper‐based and online information are not always up to date and often fail to consider individual characteristics such as educational level, age, or health literacy. These concerns align with those reported in previous studies which similarly identified a lack of practical tailored education that builds patients' confidence and skills in managing their condition (Griva et al. 2013; Lee et al. 2021; Lightfoot et al. 2022). Our findings also revealed an overlap between this issue and the broader theme of patient individuality, in that the overload of generic, paper‐based information fails to consider the diverse needs of patients. While some participants found paper‐based materials and websites helpful for improving kidney‐related knowledge, they maintained that many of these are not appropriate for populations with limited health or digital literacy and are not tailored to individual needs to self‐manage their CKD. These insights reinforce the need for tailored self‐management education and ongoing support that responds to each patient's capabilities and context based on the patient's preferences, rather than relying solely on standardised materials (Escudero‐Lopez et al. 2024; Lunardi et al. 2023).

Our finding that disease and treatment burden can significantly hinder a patient's ability to self‐manage their CKD aligns with previous research, which highlights that patients with advanced CKD commonly experience substantial physical, particularly fatigue, and mental health burdens (Guerra et al. 2021; Lopez‐Vargas et al. 2014). Hence, it is vital to differentiate CKD patients' passive attitudes, disease acceptance or physical/mental health burden that require a more urgent assessment and treatment. Mental health burden was identified in the present study as one of the most common barriers to active self‐management. The emotional burden associated with CKD progression is not always diagnosed and managed in a timely manner by clinicians (Gao et al. 2025; Lopez‐Vargas et al. 2014). Findings from our study highlighted the adverse effect of suffering from advanced CKD, changing nearly every aspect of daily living, including employment, social life, diet and exercise, while at the same time adding an extra financial burden (income and insurance), often causing emotional distress. Our findings are consistent with prior studies highlighting the risk of mental health deterioration due to the overwhelming life changes imposed by CKD (Guerra et al. 2021; Lightfoot et al. 2022; Lopez‐Vargas et al. 2014). Addressing disease and treatment burden holistically is thus crucial to supporting patients' capacity to engage in self‐management.

Access to healthcare team services, particularly mental health and allied health support, emerged as another key theme influencing CKD self‐management. Participants who had not yet commenced KRT reported fewer interactions with allied health professionals, such as dietitians, pharmacists, and psychologists, and identified a notable gap in mental health support. This finding is consistent with previous research indicating that patients not yet on dialysis often receive less structured and coordinated care, despite experiencing substantial disease burden (Guerra et al. 2021; Hong et al. 2017; Lightfoot et al. 2022; Majeed‐Ariss et al. 2017). The substantial physical and psychological burden of CKD and its treatments can reduce patients’ motivation and capacity to self‐manage their condition, potentially contributing to the onset or exacerbation of psychological distress, particularly in the absence of appropriate healthcare support (Gao et al. 2025; Lightfoot et al. 2022; Lopez‐Vargas et al. 2014; Magadi et al. 2022). If effective healthcare can alleviate symptom burden, patients may feel more able to undertake self‐management tasks (Allen et al. 2024; Magadi et al. 2022; Schrauben et al. 2018). While a recent systematic review has highlighted the importance of tailored psychological interventions before dialysis initiation though a patient‐centred care approach (Byrne et al. 2023), our findings highlight that such support is not routinely available. Participants and clinicians alike recognised that effective symptom management and psychosocial support can enhance patients' motivation and capacity to engage in self‐management tasks. A further key theme identified in our study was the importance of a patient‐clinician relationship that encompasses both emotional and informational components, including mutual trust, empathy, respect and acceptance for effective communication. Participants emphasised the value of long‐term relationships with clinicians, where trust, empathy and respect had been established over time. Conversely, ineffective communication, especially the use of jargon, was seen as hindering patients from participating in the shared decision‐making process and self‐managing their CKD. The patient‐clinician relationship is central to patient‐centred care, fostering trust, mutual respect and shared decision‐making, as well as an understanding of the patient's healthcare needs and preferences (Lightfoot et al. 2022). Although clinicians recognised the importance of relational care, they also described structural and time‐based constraints that limited their ability to consistently provide personalised support. This divergence highlights the need for system‐level changes, alongside communication training, to support meaningful clinician‐patient engagement.

Clinician teaching strategies that promote critical and reflective thinking, skills and confidence for patients to learn how to effectively self‐manage their CKD was the final key theme identified as essential for effective self‐management in our study. This aligns with previous research suggesting that patients who possess the skills and confidence to shape more productive interactions can achieve effective communication with their clinicians and are more adept at getting their healthcare providers to be responsive to their needs (Lightfoot et al. 2022). These findings align with evidence demonstrating that education, coaching, and/or counselling can enhance activation and self‐management behaviours in CKD (Allen et al. 2024; Lightfoot et al. 2022; Lunardi et al. 2024).

Patients with CKD require accessible and responsive clinicians who can offer clear, personalised guidance, not only to educate them about their illness but also to motivate and support them, particularly during periods of emotional distress, demotivation, frustration or mental health decline (Escudero‐Lopez et al. 2024; Lunardi et al. 2024). This finding aligns with previous systematic reviews highlighting the critical role of clinicians in building patients' capacity and equipping them with the necessary resources to overcome self‐managing barriers (Escudero‐Lopez et al. 2024). Effective self‐management of CKD is grounded in a dynamic partnership, where both patient and clinician contribute their respective expertise (lived experience and clinical knowledge) to collaboratively navigate health challenges (Griva et al. 2013). However, systemic barriers such as clinician shortages, time constraints, and insufficient training in behaviour change strategies or motivational interviewing can significantly limit the support patients receive (Årestedt et al. 2020; Hong et al. 2017; Lo et al. 2016). Consistent with existing literature, our study found that poor clinician‐patient relationships undermine shared decision‐making, reduce patient trust in the healthcare system, and diminish motivation to adhere to clinician recommendations (Escudero‐Lopez et al. 2024; Ladin et al. 2017). Incorporating routine assessment of patient activation in clinical settings may help identify patients who would benefit most from targeted, patient‐centred interventions. A tailored, activation‐guided self‐management program could provide a scalable, effective approach to addressing systemic limitations and improving CKD outcomes.

## 5 Implications for Clinical Practice

Our findings point to several practical opportunities to strengthen support for CKD self‐management. First, routinely assessment of patient readiness and activation (e.g., confidence, information needs, emotional burden) could enable education to be better tailored, rather than relying primarily on standardised printed resources. Second, earlier and stage‐appropriate access to multidisciplinary input (dietician/pharmacist and psychosocial support) may help address perceived gaps in guidance prior to KRT initiation. Third, fostering relational care by minimising jargon, using teach‐back, and incorporating patient goals into shared decision‐making may strengthen trust and patients' perceived control. Finally, embedding routine screening and referral pathways for distress may address the emotional burden which can otherwise reducethe capacity to engage in self‐management behavior.

## 6 Strengths and Limitations

A key strength of this study is its consumer‐led design, with people living with CKD involved throughout the research process. This enhanced cultural authenticity and ensured that findings reflected lived experience. Additional strengths include the use of mixed consumer–clinician focus groups, which enabled exploration of shared and divergent perspectives, and the use of reflexive thematic analysis supported by a multi‐disciplinary team. Additionally, the diversity of participants in terms of age and socio‐economic background further enriched the findings. Limitations include the composition of the consumer sample. Only one participant was not yet receiving KRT at the time of data collection, all other participants had progressed to receiving KRT therapy. As such, insights relating to pre‐KRT self‐management are primarily retrospective and should be interpreted cautiously. Future research should prioritise the inclusion of patients currently in earlier stages of CKD to more fully capture prospective pre‐KRT self‐management needs. Additional limitations include the single‐site South Australian context, which may limit transferability to regions with different service structures. Mixed groups (consumer and clinician) may have introduced some degree of social desirability bias, although this was mitigated by consumer co‐facilitators and explicit group agreements. While the sample captured variation in CKD stage and background, men were over‐represented, and very late‐stage CKD participants were under‐represented, which may limit insight into advanced‐stage experiences. As with all qualitative research, findings reflect interpretive co‐construction between participants and researchers, although this was strengthened by member checking and reflexive practice.

## 7 Conclusion

This study identified six themes to describe what helped and hindered patients from actively engaging in CKD self‐management, informed by participants' retrospective reflections on earlier stages of their CKD journey. Within these themes, participants identified several enablers including a positive mindset to overcome the health changes associated with their CKD progression and the importance of having the caregiver's support. Conversely, effective communication between clinician ‐patient using a person‐centred care approach based on trust and good communication emerged as essential to empower patients to take a more effective role to self‐manage their CKD. Key patient barriers identified in this study included the mental, physical and treatment burden associated with the CKD journey and insufficient communication with didactic advice by clinicians. This study provides new insights into the emotional, informational, relational, and system‐level factors shaping CKD self‐management among adults not yet receiving KRT. A consumer‐led approach revealed how personal readiness, emotional burden, service fragmentation, and the quality of patient–clinician relationships influence patients' ability to manage their condition. Improving self‐management support will require tailored education, integrated psychosocial care, and organisational changes that strengthen relational and communication‐based practices.

## Author Contributions

L.E.L. conceived the study and designed it in collaboration with M.B., D.B., R.B., D.M., R.L., E.J., renal consumers with over 20 years of CKD lived experience, and P.N.B., L.A.M., R.K.L.L., and S.M., highly qualified. R.B., E.J., D.M., P.N.B. and L.A.M. conducted the interview. L.E.L. and R.L. transcribed the interviews. L.E.L., P.N.B. and L.A.M. coded the data and R.L. reviewed the data. Initial drafts of the manuscript were written by L.E.L., which was reviewed and edited by L.A.M., P.N.B., R.L., D.D., A.B. and S.J. All authors contributed to the study and approved the final manuscript.

## Ethics Statement

The study protocol was approved by the CALHN research committee (reference number 19031) and the institutional review board at the University of South Australia (Human Research Ethics Application ID 206246). This study was registered with the Australian New Zealand Clinical Trials Registry (ANZCRT) # 12625000136404.

## Conflicts of Interest

The authors declare no conflicts of interest.

## Supporting information


**Supplementary Material 1.** Consolidated Criteria for Reporting Qualitative Research (COREQ) Checklist.

Supplementary_Material_Interview_Guide.

## Data Availability

The data supporting the findings of this study are available from the corresponding author upon reasonable request. The data are not publicly available due to privacy or ethical restrictions.
