# Supplementary material for: “Understanding Chronic Kidney Disease Self‐Management Barriers and Facilitators: A Consumer‐Led Qualitative Study”
Source: J Ren Care. 2026 Mar 5;52(1):e70055. doi: 10.1111/jorc.70055 (PMC12961917; doi:10.1111/jorc.70055)
Supplement: Supplementary file 2 — Supplementary_Material_Interview_Guide. [file JORC-52-0-s002.docx]

**2. Supplemental Material**

Interview Question Guide

Focus Group running sheet.

Introduction: This is a study intend to learn the consumers’ experience regarding what helped and what hindered you in your own management of their health related to your kidney disease. We are also interested in the health care professionals’ views and what their experience has been in promoting self-management. We have some observers in the room who will not be contributing at all as they will be simply observers. There is a participant demographic sheet in front of participants that we ask you to complete.

This study is totally voluntary; participants are free not to respond to any questions and can leave at any time. If the questions cause any discomfort or concerns, please don’t hesitate to go to the principal investigator (PI) (LL) and she will assist you. You will note that we will be recording this. The recordings will be transcribed into a word document and the audio recording will be destroyed to avoid identification. We remind you that the information here will be confidential and we ask you to agree not to discuss other peoples’ responses after we leave today. We will be on a first name basis tonight, and we won't use any first or last names in our reports or publications. Although it may be difficult can we try not to mention doctors or nurses by name, just say my nephrologists or my nurse, etc. Everyone’s experiences and opinions will be valued, and we will attempt to provide equal time for each participant to respond and if someone takes longer, we may have to interrupt to allow others to respond. The questions that you have been emailed by the PI will be up on the screen, however, sometimes we may ask additional questions if this helps us achieve the major aims of the session.

Do you have any questions before we start?

1. We have talked about some of the things we like to do. How does looking after your health help you enjoying these activities? (All)
2. What does self-management of your health care mean to you? (All)
3. Can you tell me a good experience you have had in your kidney health journey? (Consumers)
4. Can you tell me about a bad experience you have had in your kidney health journey? (Consumers)
5. What have health professionals done (or not done) to help you manage your own care? (consumers)
6. What about family and friends? What involvement have they had in helping you manage your own care? (Consumers and HCPs)
7. What helpful education have you received about managing your own health care? (Consumers)
8. What education do you provide to enhance self-management? (HCPs)
9. Is there anything that has stopped you from managing your own care? (all)
10. How would you like a self-management program delivered?
11. Remembering the aim of today is there anything else you wish to share?
12. As we are drawing to an end to our 2 hours we would like to thank you for your time. As a reminder we remind you that the information here will be confidential and we ask you to agree not to discuss other peoples’ responses after we leave today.
